# Lignin-Based Carbon Nanofibers as Electrodes for Vanadium Redox Couple Electrochemistry

**DOI:** 10.3390/nano9010106

**Published:** 2019-01-16

**Authors:** Jose Francisco Vivo-Vilches, Alain Celzard, Vanessa Fierro, Isabelle Devin-Ziegler, Nicolas Brosse, Anthony Dufour, Mathieu Etienne

**Affiliations:** 1 Laboratoire de Chimie Physique et Microbiologie pour les Matériaux et l’Environnement, UMR 7564 CNRS and Université de Lorraine, F-54600 Villers-lès-Nancy, France; 2 Institut Jean Lamour, UMR 7198 CNRS and Université de Lorraine, F-88000 Épinal, France; alain.celzard@univ-lorraine.fr (A.C.); vanessa.fierro@univ-lorraine.fr (V.F.); 3 Laboratoire d’Etude et de Recherche sur le MAteriau Bois (LERMAB), Université de Lorraine – Campus Aiguillettes, F-54506 Vandœuvre-lès-Nancy, France; isabelle.ziegler@univ-lorraine.fr (I.D.-Z.); nicolas.brosse@univ-lorraine.fr (N.B.); 4 Laboratoire Réactions et Génie des Procédés, UMR 7274 CNRS and Université de Lorraine, F-54000 Nancy, France; anthony.dufour@univ-lorraine.fr

**Keywords:** electrospinning, lignin, carbon nanofibers, vanadium electrochemistry

## Abstract

Three different types of lignin (kraft, organosolv and phosphoric acid lignin) were characterized and tested as precursors of electrospun nanofibers. Polyethylene oxide (PEO) was added as a plasticizer and dimethyl formamide (DMF) employed as a solvent. It was found that the molecular weight of lignin was the key parameter to understand the differences of the mechanical stability of the resultant fiber mats. In the case of kraft lignin (KL), the influence of some changes in the synthetic process was also tested: applied voltage, pretreatment in air or not, and the addition of a small amount of Ketjen black. After pyrolysis in nitrogen flow, the obtained carbon nanofibers (CNFs) were characterized by different techniques to analyze their differences in morphology and surface chemistry. Vanadium electrochemistry in 3M sulfuric acid was used to evaluate the different CNFs. All fibers allowed electrochemical reactions, but we observed that the oxidation of V(II) to V(III) was very sensitive to the nature of the raw material. Materials prepared from kraft and phosphorus lignin showed the best performances. Nevertheless, when 1 wt.% of Ketjen black was added to KL during the electrospinning, the electrochemical performance of the sample was significantly improved and all targeted reactions for an all-vanadium redox flow battery were observed. Therefore, in this work, we demonstrated that CNFs obtained by the electrospinning of lignin can be employed as electrodes for vanadium electrochemistry, and their properties can be tuned to improve their electrochemical properties.

## 1. Introduction

Lignin is produced as a waste product from the cellulose pulping process, which yields more than 60 megatons per year of this byproduct, which is one of the most abundant polymers on Earth [1]. While most lignin is employed as a fuel, other applications have been explored in the literature to obtain value-added products such as chemicals [2,3]. Nevertheless, this is a quite difficult process due to the complexity of the lignin molecule, which makes the separation of all the chemicals generated during the catalytic cracking of this polymer difficult. Furthermore, there are several types of lignin depending on the source and the extraction protocol, which affects the reproducibility of the process regarding the yield for different products. Therefore, over the last decades, research efforts have focused on exploring other ways to valorize the lignin, and one of the most important is the synthesis of carbon materials such as activated carbons [4,5], hierarchical porous carbons [6,7], and carbon nanofibers [8,9,10,11,12].

Carbon nanofibers are mainly produced by the carbonization of polyacrylonitrile (PAN) [13,14,15,16]. While this polymer can be synthetized in large amounts nowadays, its corresponding monomer, acrylonitrile, is obtained from propylene, a byproduct from oil refining, and therefore it is not a renewable precursor. The cost of lignin is lower than that of PAN and the process to get the carbon nanofibers is more environment-friendly, since hazardous gases and volatile compounds emissions are avoided. While different types of spinning techniques were developed to produce fibers of different sizes from lignin, electrospinning appeared as a sound alternative due to its versatility [8,9,10,11,12,17]. Moreover, to date, it is the only technique which allows the control of the fiber diameters at both nanometer and micrometer scales.

Electrospinning set-ups are basically composed of a pump which contains a syringe with the precursor suspension, a high-voltage source, and a metallic plate where the sample is collected [11,12,18]. The needle of the syringe is connected to the collector through the high-voltage source, so the suspension transitions from the syringe to this metallic plate while the solvent evaporates during the process. The diameter of the fiber depends on the so-called Taylor’s cone formation, and so it is related to different experimental variables such as the nature of the solvent, viscosity of the suspension, flow of injection, needle diameter, needle-to-collector distance, and applied voltage [1,14,18]. This fact increases the versatility of the process, allowing fibers of very different sizes to be obtained and, as long as these variables are strictly controlled, the process is reproducible. Furthermore, modifications such as coaxial [12,15] or triaxial [19] configurations can be implemented, adding even more possibilities to the technique. Fibers can be produced by electrospinning from very different precursors such as inorganic mixed oxides (for instance, indium tin oxide (ITO) [18]), synthetic polymers such as PAN [13,15] and, of course, natural polymers such as polysaccharides, cellulose or lignin [9,11,12,17,20,21,22,23].

Regarding vanadium electrochemistry, there has been an increasing interest over the last years in finding new environmentally-friendly energy sources for electrical energy production [6,24,25,26,27,28]. The problem of most renewable energy sources such as solar or wind is their intermittent production, and so electric energy is not necessarily generated when needed for consumption and, therefore, needs to be stored [6,29]. In such a way, there is an increasing interest in technologies that permit the storage of a great amount of energy and release it whenever needed, such as supercapacitors [30,31,32] and batteries [8,26,33]. The redox flow battery is a technology that allows the storage of electrical energy in liquid electrolytes from separated tanks that are pumped to stacks of electrochemical conversion cells, eliminating self-discharge, reducing the size of electronic device, increasing the flexibility of the design and the long-term stability of the system [29,34,35,36]. From the different electrolytes that have been proposed, all-vanadium redox flow batteries are the most attractive to date, since they contain the same electrolyte for the catholyte and the anolyte, avoiding the possibility of cross-contamination through the membrane which separates both compartments [29,37,38,39].

In this work, a series of lignin-derived carbon nanofibers were obtained by electrospinning, using polyethylene oxide (PEO) as a plasticizer and dimethylformamide (DMF) as a solvent. Three different types of lignin (kraft lignin, organosolv lignin and phosphoric acid lignin) were used as precursors, and electrospinning conditions were adjusted. The three types of lignin were characterized, as well as the obtained carbon nanofibers, and the cross-property relationships between the obtained fibers and lignin characteristics were investigated. Once pyrolyzed, the carbon nanofibers (CNFs) were tested as electrodes, employing an electrolyte with dissolved V^3+^ and VO^2+^ species. Their performances as promoters of vanadium oxidation and reduction reactions were related to the CNF surface chemistry and morphology.

## 2. Materials and Methods

### 2.1. Reagents

Vanadium trichloride (VCl_3_, 97 wt.%), vanadium oxysulfate hydrate (VOSO_4_·*x*H_2_O, *x* = 3–5, 97 wt.%), sulfuric acid (H_2_SO_4_, 95–98 wt.%), polyethylene oxide (PEO, MW400,000) and dimethylformamide (DMF, 99.8 wt.%) were supplied by Sigma-Aldrich (Lesquin, France).

### 2.2. Origin of Lignins

Kraft lignin (KL) was supplied by Borregaard Lignotech (Paris, France), extracted from coniferous wood according to the standard kraft process. Phosphoric acid lignin (PL) was produced from hardwood using concentrated phosphoric acid (H_3_PO_4_, 70 wt.%) pre-treatment. Ethanol organosolv lignin (EOL) was extracted from beech wood according to a procedure already optimized in the literature to maximize its yield [40,41,42]. Briefly, milled wood was introduced into a high-pressure stirred reactor of 10 L (Grayel et Fils—model 15610, Saint Genis Laval, France) containing an ethanol/water solution (65/35 % v/v) and H_2_SO_4_ (1.5% w/w (sulfuric acid/dry beech wood)) as a catalyst, and heating up to 195 °C for a residence time of 80 min. The dry-wood-to-ethanol solution proportion was 1 to 10 (w/v). After cooling to room temperature, the black liquor was filtered to separate the pulp, and then distilled water was added to the liquor (3/1 v/v) to precipitate EOL. EOL was then separated by centrifugation (2000 rpm) and dried at 40 °C.

### 2.3. Lignin Characterization

The three types of lignin were characterized by different techniques. Chemical groups were qualitatively analyzed by Fourier-transform infrared spectroscopy (FTIR Spectrum 2000, Perkin Elmer, Waltham, MA, USA), assigning the different signals according to the bibliography in [43,44]. To analyze the purity, the Klason lignin protocol was applied [3,42]. Solid-state ^13^C nuclear magnetic resonance (Bruker Avance II spectrometer operating at 9.4T, corresponding to a ^13^C Larmor frequency of 100.6MHz, Billerica, MA, USA) was also performed to analyze the amounts of different functional groups and the purity of lignin. The molecular weight distribution was obtained by means of size exclusion chromatography (SEC). For that purpose, the three types of lignin were first acetylated using a protocol described in the literature [41,42,44]. Samples of acetylated lignin were thus dissolved in tetrahydrofuran (THF, 1 mg/mL), filtered through a 0.45 μm filter (Whatman, Maidstone, UK), and then 20 µL of the solution was injected in the chromatograph (Shimadzu Prominence HPLC system, Kioto, Japan) equipped with a UV detector at 280 and 254 nm. A column sequence consisting of a Shodex GPC KF-G guard column (Munich, Germany), a Shodex GPC KF-806L column (Munich, Germany) and a Phenogel™ 5 µm 100A column (Le Pecq, France) operating at 30 °C were used to eluate lignin with a THF flow rate of 1 mL min^−1^. Standard polystyrene samples were used to plot a calibration curve. Data were collected and analysed with LabSolutions LCGC (Shimadzu, Kioto, Japan) software.

### 2.4. Electrospinning of Lignin and Thermal Treatments

Suspensions of the three types of lignin were prepared as follows. Polyethylene oxide was employed as a plasticizer in the appropriate lignin/PEO ratio (90/10, 20 wt.% in DMF) and dissolved at 60 °C in DMF for two hours under magnetic stirring. Then, lignin was added to the solution and suspended by means of a heated ultrasonic bath (70 °C for 3 h). The suspension was then removed from the bath and left to cool down to room temperature overnight, under stirring. Prior to using it, lignin was re-suspended in an ultrasonic bath without heating for 30 min.

In order to obtain the carbon nanofibers from lignin, a homemade electrospinning set-up (Figure S1) was employed similar to the one described elsewhere for preparing electrospun ITO fibers [18]. This horizontal set-up is formed by a syringe pump (KdScientific, Holliston, MA, USA) in which the syringe containing the precursor solution was placed. The syringe needle was a 21G needle (inner diameter 0.8 mm) which was connected to a stainless-steel collector (28 × 28 cm^2^) through a high-voltage power source (Iseg, Radeberg, Germany). For all experiments, the pumping rate was fixed at 3 L min^−1^, the deposition time at 3 h, and the needle-to-collector distance at 10 cm. The voltage was optimized in the case of fibers obtained from KL by changing it from 7 to 9 kV, whereas the rest of the precursors were processed at 9 kV. In the case of KL, the influence of adding a small amount (1 wt.%) of conductive carbon (Ketjen black) was also tested. Once electrospinning was finished, the obtained materials were stripped from the collector.

In the case of KL, some mats were heated in air (200 °C, 2 h, 1 °C min^−1^) in order to analyze the influence of such pretreatment. This protocol was also followed for the electrospun fibers obtained from PL, but not in the case of the EOL ones, since these corresponding fibers did not survive it. Whether pretreated in air or not, all the samples were then pyrolyzed under a nitrogen atmosphere (first ramp: 10 °C min^−1^ up to 250 °C, 1 h; second ramp: 5 °C min^−1^ up to 1000 °C, 1 h) to obtain carbon nanofibers (CNFs).

### 2.5. Electrospun Carbon Nanofiber Characterization

The obtained CNFs were characterized in terms of their microstructure by scanning electron microscopy (SEM) with an XL30 S-FEG (Philips, Amsterdam, Netherlands) apparatus operating at 3.0 kV. SEM micrographs were analyzed to obtain information about the samples’ morphology and nanofiber diameters. Raman spectroscopy was also employed, using a confocal Raman microscope (inVia^®^ Qontor with a Peltier-cooled CCD camera, Renishaw, Wotton-under-Edge, UK) equipped with a 532 nm laser (irradiance < 5 kW cm^−2^), a 50× objective with 0.55 of numerical aperture, a 1200 lines·mm^−1^ grating, and a spectral resolution of about 3 cm^−1^ (10 acquisitions of 4 seconds). Each sample was analysed at ten different points to check the possible presence of heterogeneities at the surface and/or significant deviations along the materials’ surface. The results for each sample were averaged to obtain the relative intensities for the G and the D-band (*I*_G_ and *I*_D_) characteristic of the carbon materials.

### 2.6. Electrochemical Characterization

Vanadium electrochemistry was analyzed by cyclic voltammetry (CV) with a potentiostat (SP 50, Bio-Logic Instruments, Seyssinet-Pariset, France). A three-electrode setup was used in which a Ag/AgCl 3M KCl electrode acted as the reference (RE), a graphite rod was used as the counter-electrode (CE), and CNF as the working electrode. A glass electrochemical cell with a PVC base screwed to a metal plate was used. The PVC base had an aperture of 5 mm leaving the CNFs exposed to the electrolyte. The material was placed over a collector (a Sigracet bipolar plate BBP4, which was tested by using it directly, as working electrode (WE) and no signal was found) and an aluminum foil was used to make the external connection with the potentiostat. The cell was covered with a cap with holes for the RE, the CE and the tube carrying N_2_ gas.

CV curves were recorded at 5 mV s^−1^ in a potential window ranging from −1.0 to +1.5 V. The electrolyte solution for CV experiments consisted of a 0.15 M V(III)/V(IV) stoichiometric mixture in 3 M H_2_SO_4_. To get the electrolyte, a stock solution of 1.5 M V(III)/V(IV) in 3M H_2_SO_4_ was prepared, and this solution was diluted to the desired concentration with the appropriate volume of 3 M H_2_SO_4_.

## 3. Results and Discussion

### 3.1. Lignin Characterization

**Figure 1:**
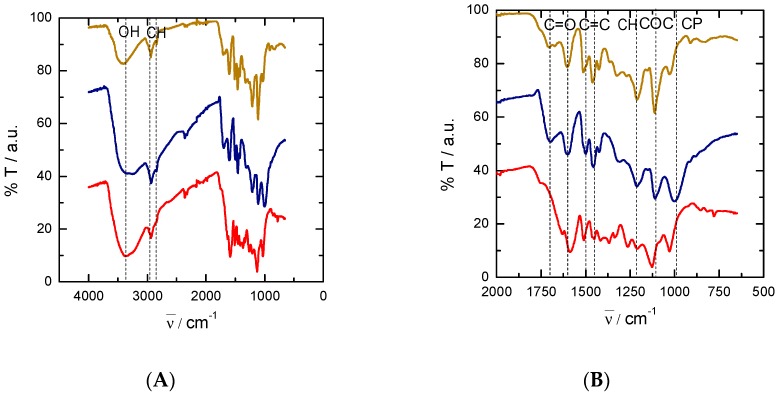
Fourier-transform infrared spectroscopy (FTIR) spectra of the three types of lignin: ethanol organosolv lignin (EOL, brown, top), phosphoric acid lignin (PL, blue, middle), and kraft lignin (KL, red, bottom). (**A**) General spectra; (**B**) Region from 500 to 2000 cm^−1^.

FTIR spectra of the three types of lignin, shifted with each other for clarity, are depicted in Figure 1. The broad band centred at 3370 cm^−1^ is assigned to hydroxyl groups, while the peaks at around 2850–2950 cm^−1^ correspond to C–H stretching vibrational modes coming from CH_2_ and CH_3_ groups (Figure 1A). The band related to the presence of hydroxyls is much weaker in the case of EOL than for KL and PL, suggesting a lower hydroxyl content in EOL. For a clearer band assignment in the region from 500 to 2000 cm^−1^, an enlarged version of the spectra is shown in Figure 1B. Signals from 1600 to 1700 cm^−1^ are related to the presence of C=O in different chemical functionalities (carbonyl, carboxylic acids or esters). From 1500 to 1600 cm^−1^, another series of peaks related to the presence of aromatic C=C bonds appears. At around 1440 cm^−1^, there are signals derived from bending modes of CH_2_ and CH_3_ groups, while bands at 1000–1200 cm^−1^ are typical of C–O–C moieties from ethers and esters. Finally, in the case of PL, a signal corresponding to phosphoric groups bonded to C is seen at around 1000 cm^−1^.

Solid state ^13^C NMR spectra of the three lignin fractions (Figure S2) present characteristic lignin signals with an intense peak at ~55 ppm, assigned to methoxyl groups of guaiacyl and syringyl units of lignin, with a signal at 60–80 ppm corresponding to the lateral chain, and a broad signal from 100 to 150 ppm corresponding to aromatic carbons. In this latter region, the intense signal at 145 ppm is attributed to oxygenated aromatics. For kraft lignin, additional signals are detected at 70–75 ppm, 85–90 ppm and 105 ppm, assigned respectively to C–2,3,5, C–4 and C–1 (anomeric) carbons of polysaccharides. This observation suggests that kraft lignin is more highly contaminated with residual sugars compared to organosolv and phosphoric acid lignins [41,42,44]. The purity of the lignin determined by the Klason protocol confirmed this result (Table 1). The lignin content is indeed lower in the case of KL (67%) compared to EOL and PL (79% and 80%, respectively). The molecular weight distribution was measured by the size exclusion chromatography (SEC) of acetylated molecules, from which the number average molecular weight ($\bar{M_{N}}$, calculated over the total number of molecules) and weight average molecular weight ($\bar{M_{W}}$, calculated considering also the weight of each molecule) were obtained (Table 1). The ratio between the two quantities is the so-called polydispersity index and increases with the width of the molecular weight distribution. In our case, the polydispersity index (P.I.) is very similar for EOL and KL (P.I. around 5–6) and is higher for PL (P.I. around 12). $\bar{M_{N}}$ and $\bar{M_{W}}$ follow the same trend: EOL is the one with the lower molecular weight, followed by KL and PL. Interestingly, we found that the molecular weight was the most important parameter defining the types of nanofibers obtained after electrospinning and pyrolysis, as described in the next section.

### 3.2. SEM Micrographs and Morphology of CNFs

**Figure 2:**
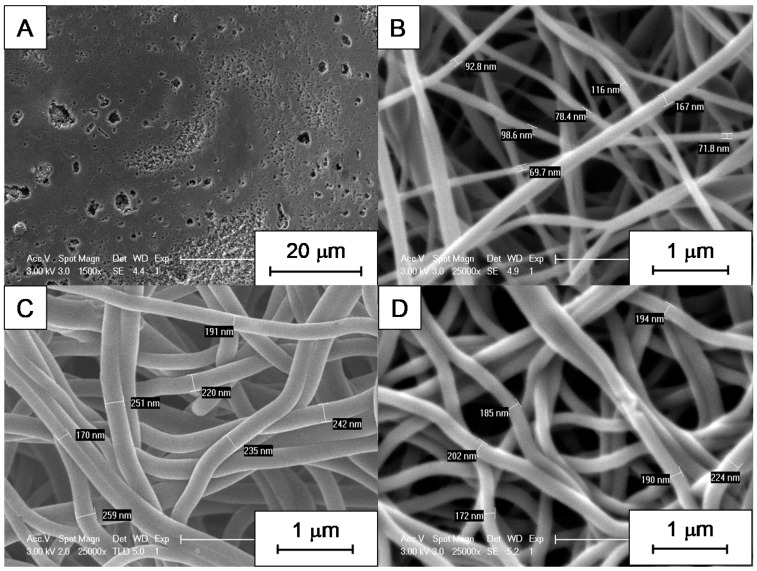
Scanning electron microscopy (SEM) micrographs of materials obtained from: (**A**) EOL; (**B**) PL; (**C**) KL and (**D**) Ketjen black added to KL. Voltage for electrospinning was 9 kV in all cases.

Variations in size were found for electrospun CNFs obtained from different types of lignin, depending on the electrospinning conditions. These variations were not only at the microscopic but also at the macroscopic level. EOL-derived fiber mats, for instance, were obtained as very small samples having poor mechanical stability, and the shrinkage induced by pyrolysis made them impossible to process for testing in vanadium electrochemistry. They did not resist the air stabilization treatment either; therefore, trials to increase their thermal stability were unsuccessful. In fact, SEM micrographs showed that there are no nanofibers in this material, which consists of a porous polymer film (Figure 2A). On the contrary, for materials obtained from PL and KL, SEM micrographs revealed that they are indeed made of carbon nanofibers (Figure 2B,C). Regarding the differences between CNFs obtained from PL and KL, the diameter of the former is much lower than that of the latter. In the case of KL, adding Ketjen black had no significant difference on nanofiber size (Figure 2D) with respect to Ketjen black-free nanofibers (Figure 2C).

**Figure 3:**
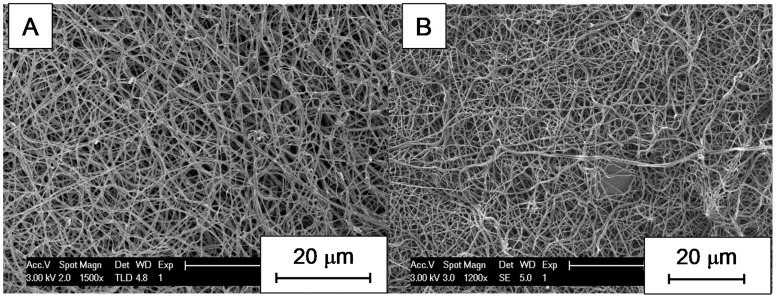
SEM micrographs of materials obtained from KL at different voltages: (**A**) 7 kV; (**B**) 9 kV.

In the case of KL, nanofibers were electrospun at 7 and 9 kV (Figure 3). No statistical difference was found in terms of the fiber diameter by analyzing SEM micrographs (the average diameter was 226 ± 22 nm for fibers obtained at 7 kV and 215 ± 33 nm for the ones obtained at 9 kV, measured for 20 different fibers along the samples; more details are provided in Figure S3). Nevertheless, the density of nanofibers increased with voltage and, therefore, the mechanical stability of the final material was improved.

### 3.3. XPS and Raman Analysis of CNFs

Not only the morphology but also the surface chemistry and the carbon structure of the CNFs depend on the type of lignin used. X-Ray Photoemission Spectroscopy (XPS) analysis (Figure S4) revealed different oxygen contents in the CNFs electrospun at 9 kV, depending on the source of lignin (Table 2). It should be recalled that the EOL-derived material was not stabilized in air, and consequently, the lowest oxygen content was found for it. Also, this lignin had a lower content of oxygenated groups according to the FTIR discussion in Section 3.1. Nevertheless, differences between KL- and PL-derived CNFs were also observed, the oxygen content being higher in the case of KL, which could be related to the higher proportion of sugars in this lignin, which is less pure than PL. A small proportion of phosphorus was also found in PL. When Ketjen black was added to KL, a significant reduction of oxygen content was found, which is not surprising since Ketjen black has no oxygen, and even though its amount in the suspension was only 1 wt.%, its fraction increased after pyrolysis given that it is already a carbon material; the weight loss thus only affects the KL-PEO part of the fibers.

**Figure 4:**
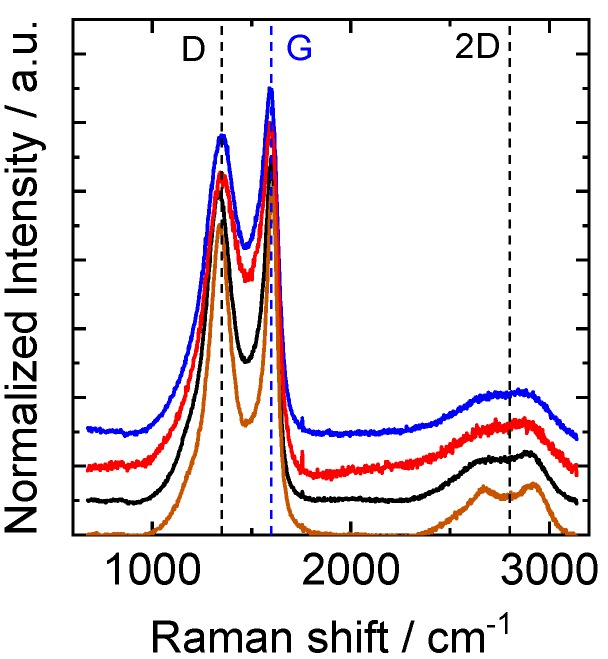
Raman spectra of the samples obtained from EOL (brown), PL (blue), KL (red) and KL loaded with Ketjen black (black). Spectra were normalized regarding the maximum intensity and shifted for clarity.

The Raman spectra of the same samples (Figure 4) were analyzed by considering the two main characteristic peaks for carbon materials: the so-called D-band, which is related to imperfections in the graphitic structure, and the G-band, which is related to graphitic carbon. The position of these two bands was very similar for all samples (Table 2). A third band, commonly assigned to an overtone of the D-band and thus called the 2D band, also appeared at around 2600 cm^−1^. The full widths at half maximum of the two peaks (G and D) were larger in the case of CNFs obtained from PL and KL than for the other two samples, even though the ratio *I*_D_/*I*_G_ remained practically the same (around 0.9). Furthermore, a doublet is found for the 2D band in the samples derived from EOL and KL loaded with Ketjen black. These two findings suggest that CNFs obtained from EOL and KL loaded with Ketjen black are more ordered than the others according to the bibliography [45,46].

### 3.4. Vanadium Electrochemistry

**Figure 5:**
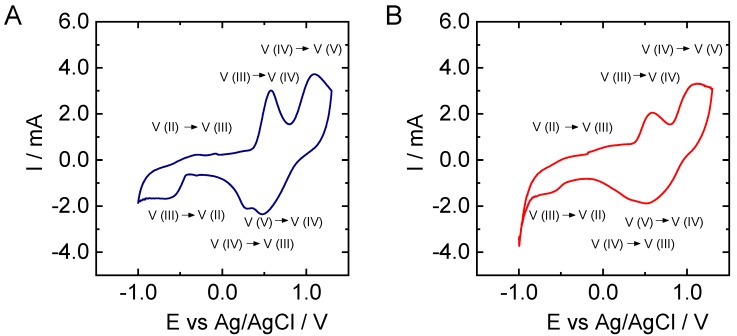
Cyclic voltammograms recorded at 5 mV·s^−1^ in 3 M H_2_SO_4_ and 0.15 M V(III)/V(IV) solution for carbon nanofibers (CNFs) derived from (**A**) PL; (**B**) KL. Both were pretreated in air and electrospun at 9 kV.

**Figure 6:**
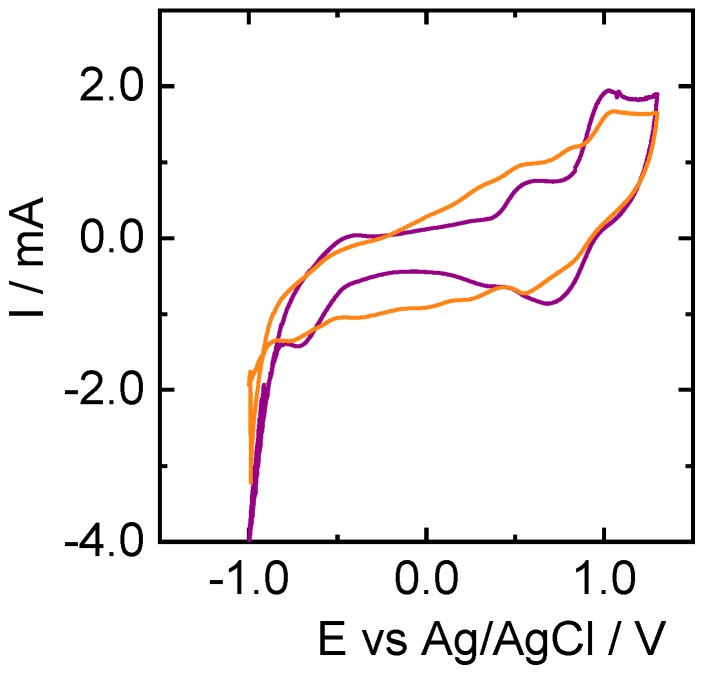
Cyclic voltammograms recorded at 5 mV·s^−1^ in 3 M H_2_SO_4_ and 0.15 M V(III)/V(IV) solution for KL-based nanofibers electrospun at 7 kV (orange) and 9 kV (purple), both without pre-treatment in air.

**Figure 7:**
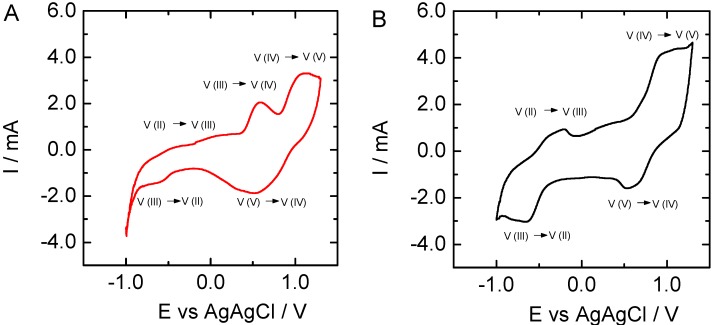
Cyclic voltammograms recorded at 5 mV·s^−1^ in 3 M H_2_SO_4_ and 0.15 M V(III)/V(IV) solution for CNFs derived from (**A**) KL; (**B**) KL loaded with Ketjen black.

Once characterized, CNFs obtained from PL and KL were employed as electrodes to test the vanadium electrochemistry (Figure 5). In the case of KL, the influence of the electrospinning voltage (Figure 6), the addition of Ketjen Black (Figure 7), and sample pretreatment on the electrochemical behavior was investigated.

When CV curves of PL- and KL-derived CNFs are compared, it becomes clear that peaks corresponding to the oxidation and reduction of vanadium species are more intense in the case of the former material. In fact, the peaks corresponding to V(III) to V (II) reduction and the reverse oxidation are only clearly visible in the case of PL-based CNFs. Nevertheless, the oxidation and reduction potentials are very similar in the two cases, giving peak potentials for V(V)/V(IV) couple of *E*_ox_ = 1.1 V and *E*_red_ = 0.5 versus Ag/AgCl reference electrode, while for V (III)/V(II) we got *E*_ox_ = −0.3 V and *E*_red_ = −0.6 V.

To analyze the influence of voltage during electrospinning, KL-based nanofibers were obtained at 7 and 9 kV. It can be observed that the signals related to vanadium reactions increased with the applied voltage at which the CNFs were obtained (Figure 6). Furthermore, signals corresponding to V(III) to V(II) reduction and its opposite oxidation are not visible in the case of KL-derived CNFs (Figure 6A), and the peak potentials separation for the V(V)/V(IV) couple is larger for this material. Regarding the influence of air treatment, differences can be observed in the electrochemical behavior when comparing the CV curve of air-pretreated KL-based CNFs prepared at 9kV (Figure 5B) with that of non-pretreated fibers (Figure 6B). For instance, the intensity of the peaks is generally higher for the sample pretreated in air. Nevertheless, in the case of the non-pretreated sample, the peak corresponding to the oxidation of V(II) to V(III) is more visible but remains lower in intensity than the corresponding reduction. Interestingly, the increase of the oxidation at −0.5 V is clearer when the relative peak intensity of the oxidation observed at 0.7 V is decreased. This latter signal can be ascribed to V(II) and/or V(III) to V(IV) oxidation.

By introducing Ketjen black during the electrospinning process, the CNFs obtained after pyrolysis were more active for vanadium electrochemistry (Figure 7), especially the oxidation of V(II) to V(III) located at about −0.5 V versus Ag/AgCl. The intensity of the signals related to vanadium species reduction and oxidation substantially increased, and potential differences between oxidation and reduction were clearly shortened from 0.6 V for V(V)/V(IV) and 0.3 V for V(III)/V(II) couples in the case of KL-PEO, to 0.4 V and 0.2 V when Ketjen Black loaded CNFs were used as an electrode. In fact, the CV curve for the sample containing Ketjen black is the only one where the signals for the V(III)/V(II) couple, especially the V(II) to V(III) oxidation, is clearly visible. Also, the signal that we ascribed before to V(II) and/or V(III) to V(IV) oxidation, at about 0.7 V vs Ag/AgCl, does not appear to be as intense for this sample. We observed before in XPS that the oxygen content was lower on the surface of these fibers with KB than for those without KB, which confirm that this carbon additive is accessible at the fiber surface, enhancing electron transfer reactions with vanadium species. In principle, this lignin-based electrode is suitable for application in all-vanadium redox flow batteries.

## 4. Conclusions

Carbon nanofibers were prepared from three different types of lignin (KL, PL and EOL) by electrospinning, using polyethylene oxide (plasticizer) and lignin suspended in DMF (solvent). Depending on the type of lignin, the carbon nanofibers were very different, and so the lignin precursors were characterized in order to understand which property was crucial for better electrospinning behavior. While the lignin purity and surface chemistry were not clearly related to the properties of the final materials, molecular weight was found to have a direct influence, with the lignin with the largest molecular weight (PL) giving the most mechanically stable nanofibers.

It was also demonstrated that carbon nanofibers obtained from lignin could serve as electrodes for vanadium electrochemistry. In this case, PL and KL presented very similar results, but those of PL were slightly better. In order to improve the electrochemical behavior of CNFs obtained from KL, different parameters were modified such as the voltage applied during the electrospinning process, air pretreatment, and the addition of a conductive filler such as Ketjen black. From these results, it was concluded that fibers obtained at 9 kV and pretreated in air exhibited the best electrochemical performance, which was further improved by the addition of KB, allowing all of the electrochemical reactions needed for all-vanadium redox flow batteries.

## Figures and Tables

**Table 1:** Purity of lignin (Klason lignin) and parameters obtained from size exclusion chromatography (SEC).

Lignin	*Purity* %	$\bar{M_{N}}$ g mol^−1^	$\bar{M_{W}}$ g mol^−1^	MW¯MN¯
Organosolv	79	266	1688	5.7
Kraft	67	317	1792	6.4
Phosphoric	80	579	7019	12.1

**Table 2:** Carbon nanotexture (Raman spectroscopy), and wt.% of C, O and P obtained by X-Ray Photoemission Spectroscopy (XPS).

Precursor	D-band cm^−1^	G-band cm^−1^	*I*_D_/*I*_G_	%C wt.%	%O wt.%	%P wt.%
EOL	1340	1605	0.91	94.4	5.6	–
PL	1347	1592	0.88	85.7	11.7	1.2
KL	1342	1592	0.88	83.8	16.2	–
KL loaded with Ketjen black	1340	1600	0.91	88.4	9.1	–

## Supplementary Materials

The following are available online at http://www.mdpi.com/2079-4991/9/1/106/s1, Figure S1: Electrospinning set-up: (**A**) syringe loaded with solution; (**B**) needle; (**C**) high voltage supply; (**D**) collector; (**E**) fibers, Figure S2: ^13^C Solid State NMR spectra of lignins: (**A**) EOL; (**B**) KL; and (**C**) PL, Figure S3: (**A**) Average fiber diameter and standard deviation obtained from the analysis of SEM images; (**B**) histogram of fiber diameter distribution for sample KL7, Figure S4: XPS survey spectra for CNFs obtained from: (**A**) EOL; (**B**) PL; (**C**) KL; (**D**) KL loaded with KB.
