# Supplementary material for: Lignin-Based Carbon Nanofibers as Electrodes for Vanadium Redox Couple Electrochemistry
Source: Nanomaterials (Basel). 2019 Jan 16;9(1):106. doi: 10.3390/nano9010106 (PMC6359536; doi:10.3390/nano9010106)
Supplement: Supplementary file 1 [file nanomaterials-09-00106-s001.pdf]

Supplementary

Lignin-Based Carbon Nanofibers as Electrodes for Vanadium Redox Couple Electrochemistry

Jose Francisco Vivo-Vilches [a,*], Alain Celzard [b], Vanessa Fierro [b], Isabelle Devin-Ziegler [c], Nicolas Brosse [c], Anthony Dufour [d] and Mathieu Etienne [a,*]

[a] Laboratoire de Chimie Physique et Microbiologie pour les Matériaux et l'Environnement, UMR 7564 CNRS and Université de Lorraine, F-54600 Villers-lès-Nancy, France

[b] Institut Jean Lamour, UMR 7198 CNRS and Université de Lorraine, F-88000 Épinal, France; alain.celzard@univ-lorraine.fr (A.C.); vanessa.fierro@univ-lorraine.fr (V.F.)

[c] Laboratoire d'Etude et de Recherche sur le MAteriau Bois (LERMAB), Université de Lorraine – Campus Aiguillettes, F-54506 Vandœuvre-lès-Nancy, France; isabelle.ziegler@univ-lorraine.fr (I.Z.); nicolas.brosse@univ-lorraine.fr (N.B.)

[d] Laboratoire Réactions et Génie des Procédés, UMR 7274 CNRS and Université de Lorraine, F-54000 Nancy, France; anthony.dufour@univ-lorraine.fr

* Correspondence: jose.vivo-vilches@univ-lorraine.fr (J.F.V.-V.); mathieu.etienne@univ-lorraine.fr (M.E.); Tel: +33-03-727-474-00 (J.F.V.-V. & M.E.); Fax: +33-03-832-754-44 (J.F.V.-V. & M.E.)

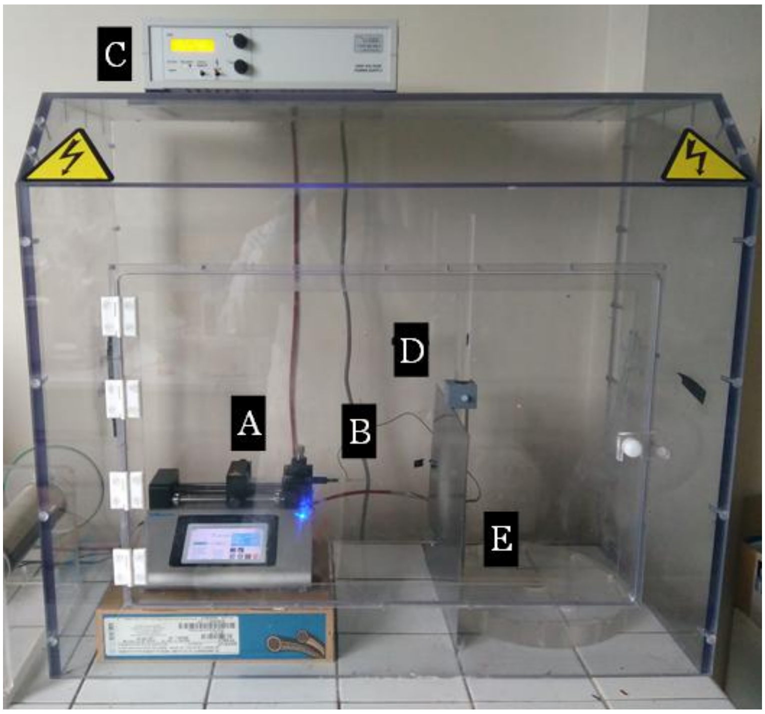

Figure S1. Electrospinning set-up: (**A**) syringe loaded with solution; (**B**) needle; (**C**) high voltage supply; (**D**) collector; (**E**) fibers.

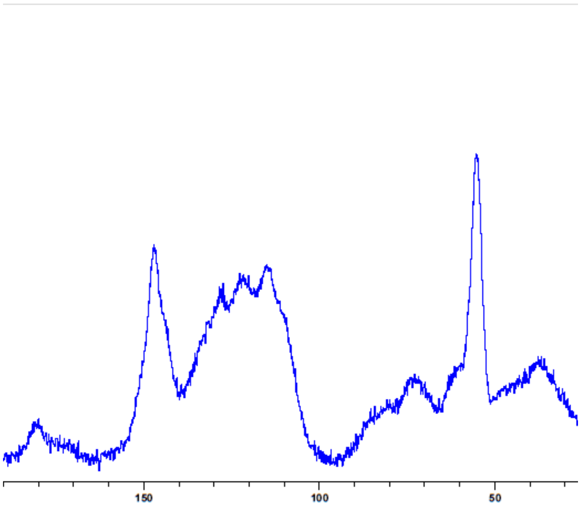

(**A**)

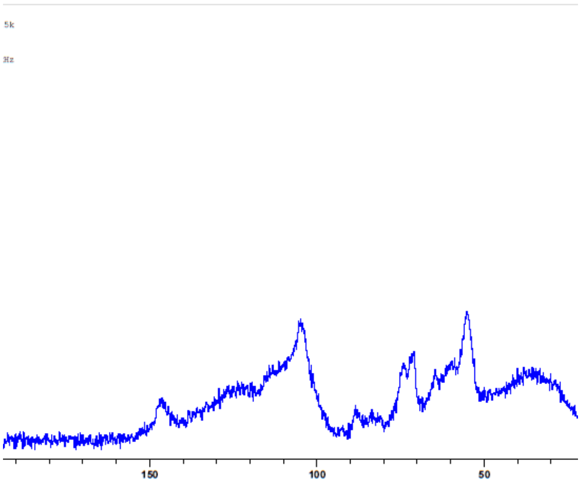

(**B**)

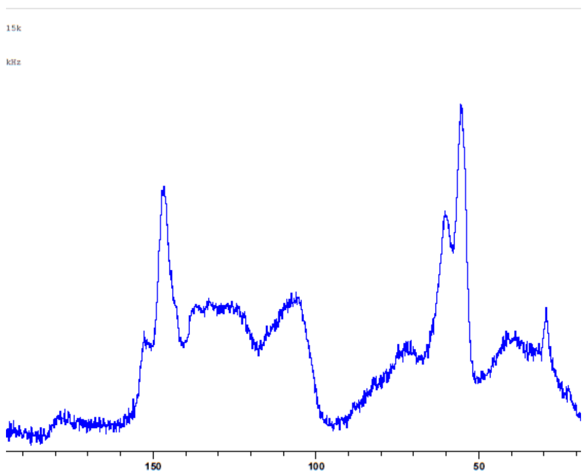

(**C**)

Figure S2. ^{13}C Solid State NMR spectra of lignins: (**A**) EOL; (**B**) KL; and (**C**) PL.

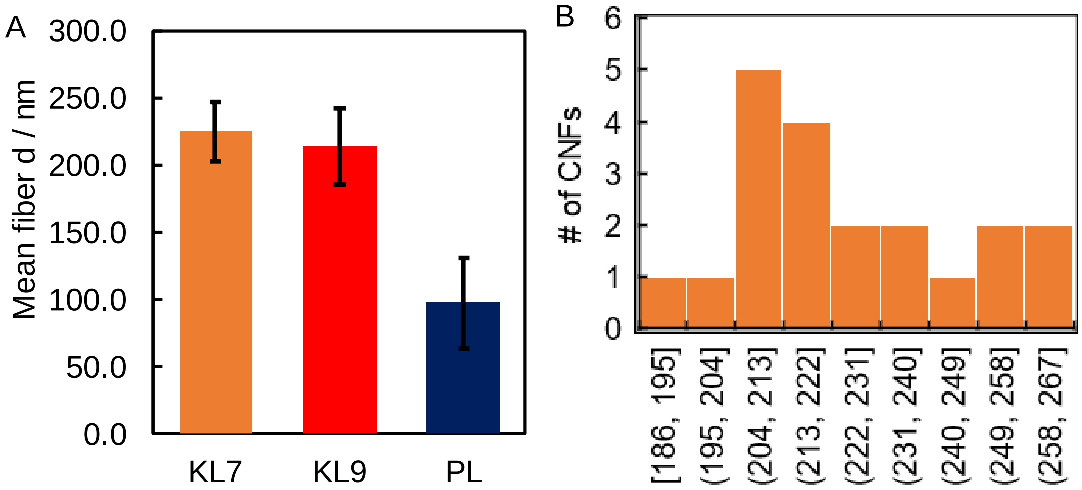

Figure S3. (**A**) Average fiber diameter and standard deviation obtained from the analysis of SEM images; (**B**) histogram of fiber diameter distribution for sample KL7.

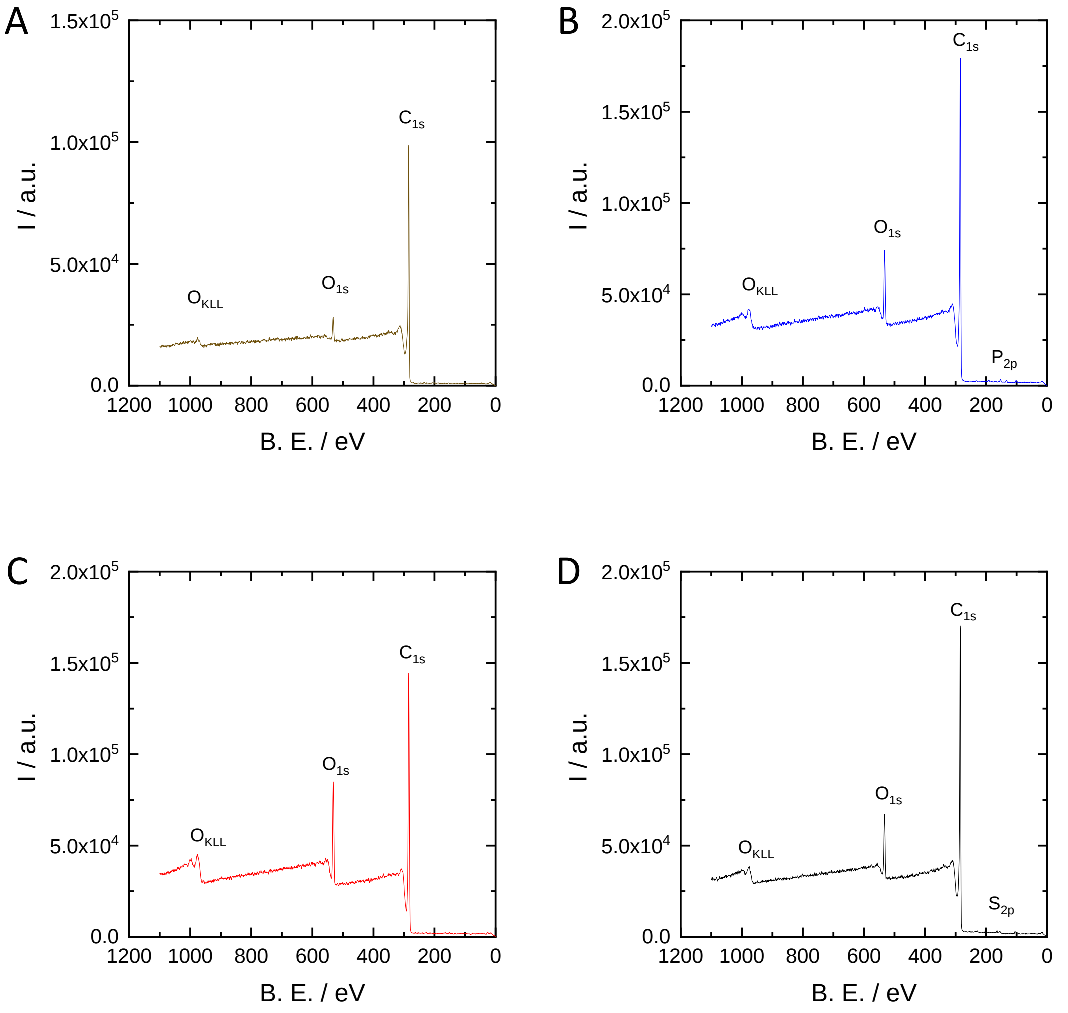

Figure S4. XPS survey spectra for CNFs obtained from: (**A**) EOL; (**B**) PL; (**C**) KL; (**D**) KL loaded with KB.
